# Development and Validation of Discriminative Dissolution Method for Metformin Immediate-Release Film-Coated Tablets

**DOI:** 10.1155/2019/4296321

**Published:** 2019-12-10

**Authors:** Ivana Mitrevska, Tina Achkoska, Katerina Brezovska, Krume Toshev, Aneta Dimitrovska, Sonja Ugarkovic

**Affiliations:** ^1^Research & Development, Alkaloid AD, Blvd. Aleksandar Makedonski 12, 1000 Skopje, North Macedonia; ^2^Faculty of Pharmacy, University “Ss Cyril and Methodius”, Mother Theresa 47, 1000 Skopje, North Macedonia

## Abstract

The purpose of this study was to develop and validate a discriminative dissolution method for the metformin film-coated tablet with immediate release of the active substance that belongs to class III of the Biopharmaceutical Classification System (BCS). Different conditions such as type of dissolution medium, volume of dissolution medium, rotation speed, apparatus, and filter suitability were evaluated. The most discriminative release profile for the metformin film-coated tablet was accomplished by using Apparatus II (paddle) and 1000 mL of phosphate buffer pH 6.8 as the dissolution medium and maintained on 37 ± 0.5°C with a rotation speed of 75 rpm. The quantification of the released active substance was performed by UV/Vis spectrophotometry, at 232 nm. Acceptance criteria for not less than 75% (Q) of the labeled content for 45 minutes were set. The dissolution method was validated according to the current international guidelines using the following parameters: specificity, accuracy, precision, linearity, robustness, and stability of the solutions, found to be meeting the predetermined acceptance criteria. A developed dissolution method has discriminatory power to reflect the characteristics of the medicinal product and is able to distinguish any changes related to quantitative formulation and can be also applied for routine batch testing.

## 1. Introduction

Dissolution tests can be used to guide the development of new formulations and to assist in proper formulation selection (selection of excipients), to assess the characteristics of the active substance (AS), and to evaluate the batch-to-batch quality and stability of the medicinal product helping in the establishment of shelf life [1– 3]. It is also commonly used as a prediction of the *in vivo* performance of a medicinal product to provide a basis for achieving *in vitro/in vivo* correlation and to minimize the need for bioequivalence studies (BE). The dissolution procedure has several distinct components. These components include a dissolution medium, an apparatus, the study design (including acceptance criteria), and the method for quantification of the released AS [4].

Fundamentally, the dissolution method should be discriminatory, and it should allow evaluating the performance of the medicinal product, particularly in monitoring AS or critical formulation parameters [5]. Metformin hydrochloride (1,1-dimethylbiguanide hydrochloride) is used in the treatment of type 2 diabetes mellitus. The active substance is highly hydrophilic and is classified as class III according to the Biopharmaceutical Classification System (BCS) with high solubility and low permeability. The active substance is ionized at physiological pH (p*K*a value is 12.4). After oral administration, metformin HCL is absorbed by the gastrointestinal mucosa. The main site of rapid release absorption is the small intestine with negligible absorption in the stomach and colon [6]. However, to develop a dissolution method, the characteristics of the AS and its behaviour in the selected test media should be taken into consideration. Moreover, the dissolution conditions must follow the *sink* conditions and the quantitation method should be specific, accurate, precise, linear, and robust [7–9]. Several dissolution methods for determination of the released percentage of the AS from the medicinal product metformin immediate-release film-coated tablets are introduced in the British (BP) and American Pharmacopoeia (USP). In the BP monograph, the recommended dissolution method for metformin immediate-release film-coated tablets uses 900 mL of phosphate buffer pH 6.8 at 37°C and baskets with 100 rpm with quantitation by spectrophotometry at 232 nm. Furthermore, the USP monograph presents three procedures for determining the percentage of released active substance depending of the dosage strengths [10, 11]. Usually, either the USP or BP methods may be suitable for generic immediate-release (IR) products, but during development, we must optimize the selected method with respect to our proposed formulation.

The objective of the present study is to develop a discriminating dissolution method for metformin film-coated tablets to support development of the medicinal product and quality control efforts for all dosage strengths. The initial part of the study was focused on the selection of suitable dissolution conditions including volume of the medium using different dissolution media, type of apparatus, rotation speed, and suitability of the filter type. Additionally, validation was performed to ensure that the developed discriminatory method accomplishes its intended purpose.

## 2. Materials and Methods

### 2.1. Material and Equipment

Metformin film-coated tablets 1000 mg, 850 mg, and 500 mg, metformin hydrochloride working standard (WS), potassium dihydrogen phosphate, and sodium hydroxide were with analytical grade. Dissolution media: pH 1.2 (HCl and NaCl), acetate buffer pH 4.5, and phosphate buffer pH 6.8 were prepared according to the directions in the European Pharmacopeia (EP) monograph.

The following instruments were used: six-station dissolution apparatus (Varian-Vankel 7025 Model: 115/230) in accordance with the USP general methods, pH meter (Mettler Toledo), hotplate stirrer (IKA C-MAG HS7), analytical balance (Sartorius CPA 225D-OCE), and UV/Visible Spectrophotometer (Varian-Model: Cary 50/60) using 10 mm quartz cells).

### 2.2. Dissolution Method

The dissolution tests on metformin film-coated tablets were performed using Apparatus I and II at 37 ± 0.5°, with a rotation speed of either 50 rpm or 75 rpm for the paddle and 100 rpm for the basket. Different dissolution media (pH 1.2 HCl and NaCl, acetate buffer pH 4.5, and phosphate buffer pH 6.8) with either 900 mL or 1000 mL were tested. Sampling aliquots of 10 mL were withdrawn at 5, 10, 15, 30, and 45 min and replaced with an equal volume of the fresh medium maintained at the same temperature. After the end of each test time, the samples aliquots were filtered through 0.45 *μ*m membrane filter (regenerated cellulose, RC), diluted with respective dissolution medium and then analyzed by the UV-Vis spectrophotometric method.

### 2.3. Preparation of Standard Stock Solutions

The stock solution was prepared by dissolving 55.55 mg of metformin hydrochloride WS in a 100.0 mL volumetric flask with the medium phosphate buffer pH 6.8. 1.0 mL of this solution was diluted to 100.0 mL with medium to obtain a concentration of 0.005 mg/mL. The solution was filtered through 0.45 *μ*m RC membrane filter.

### 2.4. Determination of Metformin Hydrochloride in the Dissolution Samples

The percentage of the released active substance from the medicinal product was determined by the UV-Vis spectrophotometric method. The UV-Vis spectra of the metformin HCL solution revealed one absorption maxima at 232 nm.

### 2.5. Filter Suitability Evaluation

Suitability of the filter type (0.45 *µ*m RC membrane filter) was determined by comparison of the absorbance obtained from six individually filtered aliquots of the standard solution of metformin hydrochloride WS (concentration of 0.005 mg/mL) with the absorbance of unfiltered standard solution.

### 2.6. Validation of the Dissolution Method

The proposed dissolution method was validated for specificity, accuracy, precision, linearity, robustness, and stability of sample solutions according to the current ICH and FDA guidelines [12, 13]. The specificity of the dissolution medium was tested by examining the peak interference from the dissolution medium and placebo in comparison with AS by an aliquot of the dissolution medium without AS (diluent), dissolution medium in which the placebo was added (placebo), dissolution medium containing AS at working concentration (standard solution), and dissolution medium with the metformin film-coated tablet (sample solution).

Accuracy of the method was tested by adding known amounts of metformin HCL WS to the placebo. Three concentrations (80%, 100%, and 120) of the theoretical working concentration were spiked, and the measurements were done in triplicate. The average recovery percentage was calculated.

The precision of the method was determined by testing the repeatability in the same day and intermediate precision (same analyst, using the same instrument) on a different day. The repeatability was tested by six replicate measurements of the absorbance of the standard solution in a concentration of 0.005 mg/mL and evaluated based on relative standard deviation (% RSD) of the results. Intermediate precision was determined with six sample solutions prepared individually using a single batch of film-coated tablets as per the test method by the same analyst, using the same instrument on a different day. Furthermore, comparison of the results variability was performed by statistical *F*-test.

Linearity was tested using six concentrations (in triplicate) of the standard solution within a concentration range from 0.00125 to 0.0075 mg/mL (25%, 50%, 75%, 100%, 125%, and 150% of the expected working concentration) and was evaluated by the linearity plot and the correlation coefficient (*r*^2^).

The robustness of the method was evaluated by variation of the three parameters: pH of the buffer (±0.2 units), rotation speed (±5 rpm), and temperature of the dissolution medium (±0.5°C). For each variation, the content of dissolved metformin HCl was calculated. The robustness of the method was examined by statistical *F*-test.

Stability of metformin HCl was evaluated using the standard solution (0.005 mg/mL) over the 48 hours test period. Sample solutions were prepared in the same dissolution media and at the same conditions as for the dissolution test. The drug concentrations observed in samples at 0, 5, 24, and 48 h were compared.

## 3. Results and Discussion

Development and validation of the discriminatory dissolution procedure was achieved following the current compendial standards in accordance with the FDA and ICH guidelines. It is worth noting that regulatory requirements at all levels of vertical follow-up (directives, regulations, procedures, recommendations, guidelines, and observations) are often strict and precise.

The dissolution procedure has several distinct components. These components include a dissolution medium, an apparatus, the study design (including acceptance criteria), and the mode of assay. All of these components must be properly chosen and developed to provide a method that is reproducible for within laboratory day-to-day operation and robust enough to enable transfer to another laboratory.

### 3.1. Selection of Dissolution Medium

During the development of the dissolution procedure, one general goal is to have “*sink*” conditions. When “*sink*” conditions are present, it is more the likely that the dissolution results will reflect the properties of the dosage form. The *sink* conditions are defined as concentrations that yield a saturation solubility of the active substance at least three times the highest dose of the active substance dissolved in the volume of the medium used for dissolution [7]. *Sink* conditions are preferred because they are more likely to result in dissolution that reflects kinetics of the active release from the dosage form rather than from solubility limitations [9]. Media deaeration is usually required and can be accomplished by heating the medium or filtering the medium (more commonly) or placing it under vacuum for a short period of time. Bubbles can cause particles to cling to the apparatus and vessel walls. On the contrary, bubbles on the dosage units may increase buoyancy, leading to an increase in the dissolution rate, or may decrease the available surface area, leading to a decrease in the dissolution rate [14].

Determination of the maximum solubility or concentration of saturated metformin HCL solution in a different dissolution media (pH 1.2 HCl with NaCl, acetate buffer pH 4.5, and phosphate buffer pH 6.8) was performed using *sink* conditions at eight concentration levels in the range of 0.0006–0.0111 mg/mL. 250 mL from each medium were transferred into flasks and placed on a magnetic stirrer with temperature at 37 ± 0.5°C in a period of 45 minutes. The results from the solubility test of metformin HCL in different proposed dissolution media are summarized in Table 1.

The performed test for achieving the *sink* condition confirmed that the metformin HCL is highly soluble in all tested media with a solubility of more than 140 mg/mL. The maximum solubility of metformin hydrochloride was achieved in phosphate buffer pH 6.8. Solubility data were used as the basis for selecting a dissolution medium for further evaluation of the medicinal product.

According to the bioavailability data, after oral administration, metformin HCl is absorbed by the gastrointestinal mucosa. Taking into consideration that the main site of absorption is in the small intestine, with physiological pH value of 6.6–7.0, the phosphate buffer pH 6.8 was selected as the dissolution medium for further dissolution testing and at the same time аs a diluent where the standard stock solution would be dissolved.

### 3.2. Selection of Medium Volume, Dissolution Apparatus, and Rotation Speed

**Figure 1:**
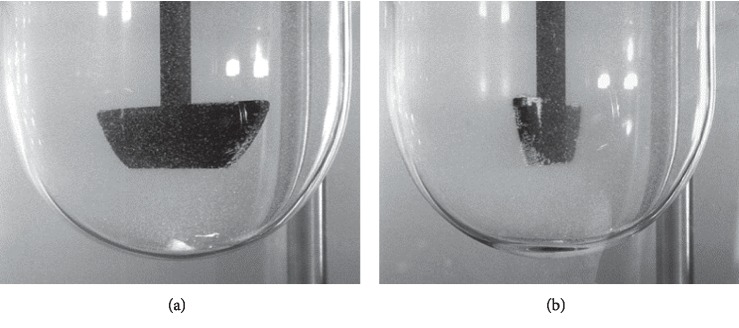
Visual observation of metformin 1000 mg film-coated tablets using paddles with rotation speed (a) 50 rpm and (b) 75 rpm.

The standard dissolution medium volumes accepted by the regulatory agencies are 500 mL, 900 mL, and 1000 mL [7]. The dissolution behaviour (variability and profile) of the dosage form itself is the best guide in choosing the volume. An effort to use one of the three standard volumes should be made to facilitate method transfer and reduce the likelihood of regulatory questions. For solid oral dosage forms, Apparatus I (basket) at 100 rpm or Apparatus II (paddle) at 50 or 75 rpm is recommended. The intent was to set a dissolution method performance that could yield data that are not highly variable and to avoid coning or mounding problems. After visual observation of the behaviour of the dosage form during the dissolution testing at 50 rpm, coning at the bottom of the Apparatus II (see Figure 1) has been noticed which leads to incomplete release of active substance and risk for obtaining variable results.

Occurrence of coning of the dosage form can be reduced by increasing the paddle speed thereby improving the results [14]. Therefore, the rotation speed of 75 rpm for the paddle was selected.

The dissolution method parameters (dissolution apparatus, rotation speed, and volume of the medium) were defined by determination of the amount of dissolved AS from metformin 1000 mg film-coated tablets in three experimental cases:

  Case 1: Apparatus I (basket) at 100 rpm, 900 mL medium
  Case 2: Apparatus I (basket) at 100 rpm, 1000 mL medium
  Case 3: Apparatus II (paddle) at 75 rpm, 1000 mL medium

**Figure 2:**
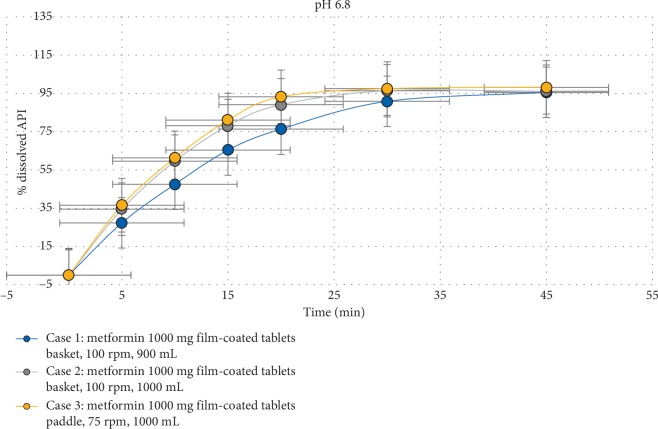
Dissolution profile of metformin 1000 mg film-coated tablets using different apparatus and volume of the medium.

Dissolution was evaluated by measuring the amount dissolved over time and carried out on six (6) tablets. Samples of 10 mL were taken after 5, 10, 15, 20, 30, and 45 minutes, and the medium was replaced to maintain the same volume. The obtained results are presented in Table 2. The dissolution profile of metformin 1000 mg film-coated tablets is shown on Figure 2.

The obtained results show that the percentage of release of the active substance in Case 1 is lower compared to the results obtained in cases 2 and 3. Furthermore, the higher variability is evident in the dissolution profile in Case 2 compared to the dissolution profile in Case 3. Therefore, the Apparatus II (paddle), 75 rpm, and 1000 mL of phosphate buffer pH 6.8 were chosen as the conditions for the dissolution method.

The dissolution procedure requires an apparatus, a dissolution medium, and test conditions that together provide a method that is discriminating, yet sufficiently rugged and reproducible for day-to-day operation and should be able to transfer between laboratories. The ideal method will have enough power to pick up changes in critical attribute that may affect the release mechanism.

**Figure 3:**
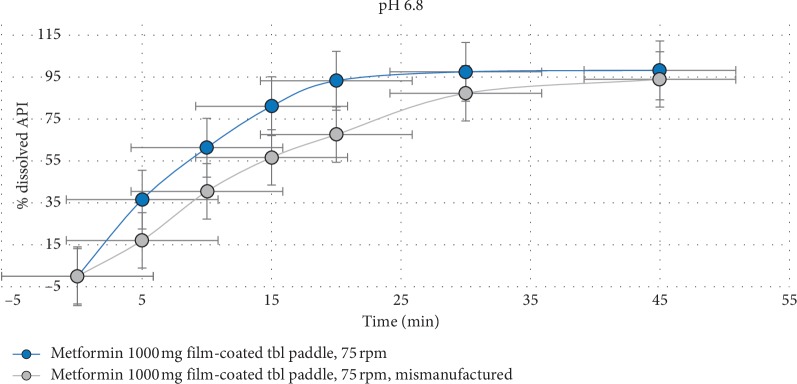
Dissolution profiles of original formulation and formulation with a deliberate change in the composition of excipients (mismanufactured).

The discriminatory power of the proposed dissolution method was confirmed by comparing the dissolution profiles for the two different formulations of metformin 1000 mg film-coated tablets: original formulation and formulation with a deliberate change in the composition of excipients (around 33% excess of magnesium stearate) [15]. Results have shown that changes of the quantitative composition of magnesium stearate (lubricant) significantly affect the rate of *in vitro* dissolution by decreasing the percent of the released active substance (see Figure 3). This concept was used to establish the factor that has the most significant influence on the dissolution rate.

**Figure 4:**
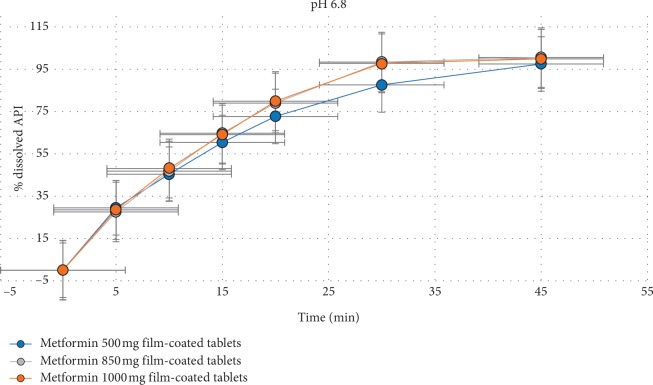
Dissolution profiles of different strengths of metformin film-coated tablets.

In order to confirm the applicability of the proposed dissolution method for all the strengths of metformin film-coated tablets (500 mg, 850 mg, and 1000 mg), the dissolution profiles in 1000 mL phosphate buffer at pH 6.8 with paddle rotation speed of 75 rpm were compared (see Figure 4).

The obtained profiles between the strengths with the developed dissolution method were considered as similar. Therefore, the experimental conditions described in Case 3 are proposed for the dissolution test in the formulation development of the medicinal product to determine the similarity of the dissolution profiles, as well as for selection of the biobatch that will be further used in the bioequivalent study. The quantitative determination of the released active substance from the medicinal product was performed using the UV/Vis spectrophotometric method at 232 nm. The acceptance criteria for not less than 75% (Q) of the labeled content for 45 minutes were set [15].

### 3.3. Filter Suitability Evaluation

The choice of a suitable filter type is important and should be experimentally justified in the early development of the dissolution method [7, 8]. The suitability of the proposed filter (0.45 *µ*m regenerated cellulose membrane filter) was confirmed by comparison of the recovery values of the concentration of metformin HCl between filtered and unfiltered standard solutions. The recovery values are within 99.23–100.15% (acceptable results are between 98.0% and 102.0%). The results indicate that the recovery is not affected when the standard solutions are filtered through the filter 0.45 *µ*m regenerated cellulose membrane filter.

### 3.4. Validation of the Dissolution Method

Method validation was performed on the highest strength of the medicinal product. The results are summarized in Table 3.

**Figure 5:**
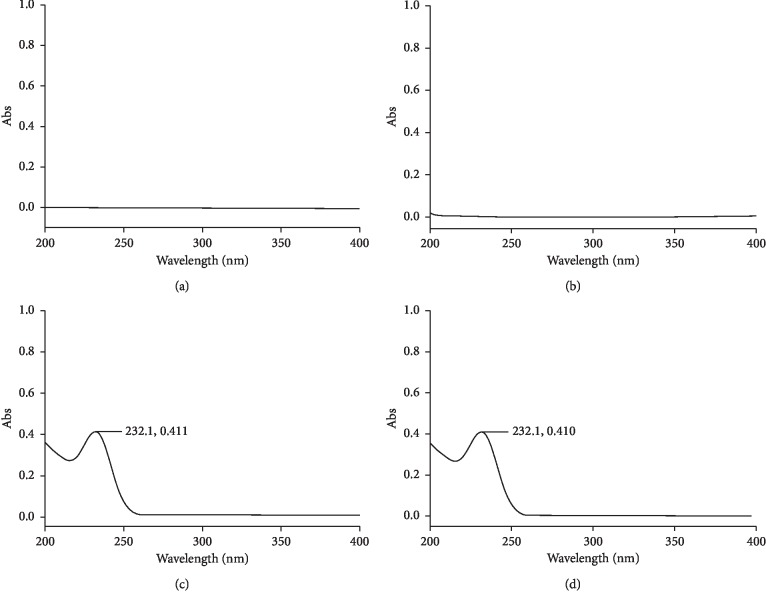
Specificity of UV spectrophotometric method for dissolution of metformin HCl. (a) Diluent. (b) Placebo. (c) Standard solution. (d) Sample solutions.

To evaluate the specificity of the dissolution procedure, it is necessary to demonstrate that the results are not affected by the placebo constituents in the medicinal product. A proper placebo should consist of everything in the formulation, except the AS. Comparison of the spectra (recorded between 200 nm and 400 nm) of the dissolution medium (diluent), placebo, standard, and sample solution shows that there is no interference between the spectra of the diluent and placebo with metformin HCl, indicating the specificity of the method (see Figure 5).

The accuracy expresses the agreement between the accepted value and the observed value. The method is accurate (the recovery values are within 95.0–105.0%, and RSD is not more than 5.0%) and precise (RSD values are less than 2%, and *F* value <5.05) [13, 15]. To assess linearity, a standard curve was constructed by plotting average absorbance versus concentration.

The correlation coefficient (*r*^2^) was greater than 0.995 for the calibration curve over the range of 25 to 150% of the working concentration, indicating a good linearity of the method.

In all the deliberately varied conditions (pH value, rotation speed, and temperature of dissolution medium), the released amount of metformin HCl from the dosage form remained unchanged, which demonstrates that the developed method is robust.

The results of the stability studies showed that the standard solution of the metformin HCL was found to be stable for 48 h at room temperature.

## 4. Conclusions

The developed dissolution method (Apparatus II with a rotation speed of 75 rpm and 1000 mL of phosphate buffer pH 6.8 as a medium) has a discriminatory power for metformin film-coated tablets and is able to distinguish any changes related to quantitative formulation of the pharmaceutical dosage form, applicable for all dosage strengths. The method can be used in the formulation development studies, as well as for the selection of biobatch for the bioequivalent study. The proposed method is specific, accurate, precise, linear, and robust and can be successfully applied for evaluation of the batch-to-batch quality and stability of the medicinal product.

## Figures and Tables

**Table 1:** Results from the solubility test of metformin HCL in the proposed dissolution media.

	pH 1.2 (HCl and NaCl)	Acetate buffer pH 4.5	Phosphate buffer pH 6.8
Solubility of metformin HCL (*n* = 2)	144.16 mg/mL	165.96 mg/mL	170.58 mg/mL

Calculated from 1000 mg/250 mL × 3 = 12 mg/mL.

**Table 2:** The obtained results of the dissolution test of metformin 1000 mg film-coated tablets using different apparatus and volume of the medium.

	Phosphate buffer pH 6.8																	
	Case 1: metformin 1000 mg film-coated tablets basket, 100 rpm, 900 mL						Case 2: metformin 1000 mg film-coated tablets basket, 100 rpm, 1000 mL						Case 3: metformin 1000 mg film-coated tablets paddle, 75 rpm, 1000 mL					
Time	5	10	15	20	30	45	5	10	15	20	30	45	5	10	15	20	30	45
Average	27.33	47.52	65.38	76.34	90.85	95.55	34.50	59.62	78.12	89.08	96.40	96.13	36.55	61.31	81.10	93.24	97.51	98.17
RSD	10.13	11.07	11.30	9.48	4.46	1.71	9.89	8.97	7.72	7.07	1.63	2.69	7.72	6.18	3.00	2.55	0.45	1.48
Min	25.11	43.02	56.90	67.33	83.79	92.37	28.45	49.83	66.42	78.44	93.31	91.14	32.78	56.85	78.19	90.29	97.07	96.39
Max	34.54	61.13	82.38	92.81	96.36	99.07	37.24	64.93	83.54	94.14	97.78	98.47	39.96	66.66	84.75	96.25	98.25	100.82

**Table 3:** Validation of the proposed dissolution method for metformin film-coated tablets.

Validation parameters	Acceptance criteria	Obtained results		
*Specificity*	No interference of diluent and placebo with metformin HCl	Conforms		

*Accuracy* Recovery level (%)	The average recovery for each level should be between 95.0 and 105.0%, and RSD should be not more than 5.0%	*Recovery (%) (average n* = *3)*	*95% confidence interval*	*RSD%*
120%		101.01%	±1.36%	0.54%
100%		101.49%	±7.15%	2.84%
80%		101.86%	±1.34%	0.53%

*Precision*	RSD < 2%			
Method precision		RSD = 1.68% (*n* = 6)		

*Precision*	*F* value < 5.05	*Day I (%)*	*Day II (%)*	
Intermediated precision	RSD < 2%	100.55%	96.13%	
		98.11%	98.02%	
		96.04%	95.63%	
		98.59%	96.45%	
		99.78%	98.61%	
		97.20%	98.79%	
		RSD = 1.68%	RSD = 1.40%	
		*F* = 1.47		

*Linearity* (within 25%–150% of the working concentration)	Correlation coefficient (*r*^2^) should be ≥0.995	*r* ^2^ = 0.99986 *y* = 0.00473 + 79.22*x*		

*Robustness*	*F* < 5.05	*Change*	*Recovery (%)*	*F value*
pH value ±2 units		6.8	—	—
		6.6	99.96%	1.09
		7.0	100.01%	1.03
Speed of dissolution tester ±5 rpm		75 rpm	—	—
		70 rpm	99.99%	1.77
		80 rpm	96.84%	2.82
Тemperature of dissolution medium ±5°С		37.0°С	—	—
		36.5°С	100.73%	3.03
		37.5°С	99.81%	2.58

*Stability of solutions*	Recovery should be between 95.0 and 105.0%	*Recovery (%)*		
Fresh preparation		—		
5 hours		100.00%		
24 hours		99.50%		
48 hours		98.60%		

## Data Availability

The data used to support the findings of this study are included within the article.
